# Progression‐free survival assessed per immune‐related or conventional response criteria, which is the better surrogate endpoint for overall survival in trials of immune‐checkpoint inhibitors in lung cancer: A systematic review and meta‐analysis

**DOI:** 10.1002/cam4.4347

**Published:** 2021-10-20

**Authors:** Guang‐Li Zhu, Kai‐Bin Yang, Si‐Qi Tang, Liang Peng

**Affiliations:** ^1^ Department of Otorhinolaryngology Head and Neck Surgery the First Affiliated Hospital of Sun Yat‐sen University Institute of Otorhinolaryngology Head and Neck Surgery Sun Yat‐sen University Guangzhou P. R. China; ^2^ Department of Radiation Oncology Sun Yat‐sen University Cancer Center Guangzhou P. R. China

**Keywords:** immune‐checkpoint inhibitors, lung cancer, progression‐free survival, surrogate endpoints

## Abstract

Progression‐free survival (PFS) has been used as a surrogate endpoint for overall survival (OS) in lung cancer trials. The pattern of response to immune‐checkpoint inhibitors (ICIs) differs from that to conventional chemotherapy, so immune‐related response evaluation criteria were proposed. This study aims at determining which PFS measure, PFS assessed per immune‐related response evaluation criteria (iPFS), or conventional criteria (cPFS), is the better surrogate endpoint for OS in trials of ICIs in lung cancer. We selected clinical trials in lung cancer that administered ICIs to at least one arm and reported both median OS and median PFS from PubMed, Embase, and The Cochrane Library. We compared the correlation between treatment effect (hazard ratio) on OS and cPFS or iPFS and the correlation between median OS and median cPFS or iPFS using weighted linear regression at trial level. We analyzed 78 ICI arms (13,438 patients) from 54 studies, including 66 arms with cPFS, seven arms with iPFS, and five arms with both kinds of PFS. We demonstrated an excellent correlation between treatment effect (hazard ratio) on OS and iPFS (*R*_WLS_^2^ = 0.91), while the correlation was moderate for cPFS (*R*_WLS_^2^ = 0.38). Similarly, the correlation between median OS and median iPFS was also strong (*R*_WLS_^2^ ranging from 0.86 to 0.96) across different phases of trials and different types of lung cancer, ICI, and treatment modalities, while it was much weaker for median cPFS (*R*_WLS_^2^ ranging from 0.28 to 0.88). In conclusion, iPFS provides better trial‐level surrogacy for OS than cPFS in trials of ICIs in lung cancer.

## Graphical abstract

It is unknown which PFS measure, PFS assessed per immune‐related response evaluation criteria (iPFS) or conventional criteria (cPFS), is better correlated with OS in practice. This meta‐analysis attempted to determine which PFS measure, iPFS or cPFS, is the better surrogate endpoint for OS in trials of immune checkpoint inhibitors in lung cancer.

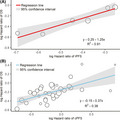

## 1 INTRODUCTION

Lung cancer ranked first worldwide in both incidence and mortality among all malignancies in 2018.^1^ For advanced or recurrent lung cancer, the prognosis is still poor. Cytotoxic drugs have limited effect on advanced or recurrent lung cancer. Over the past few years, immune‐checkpoint inhibitors (ICIs), including anti‐PD‐1, anti‐PD‐L1, and anti‐CTLA‐4 antibodies, have shown favorable efficacy in both advanced non‐small cell lung cancer (NSCLC) and extensive‐stage small cell lung cancer (SCLC).^2^, ^3^ More trials investigating ICIs in advanced lung cancer are ongoing.

Overall survival (OS) is the gold standard in the evaluation of efficacy in oncology clinical trials. Although the measurement of OS is simple and reliable, the treatment effect on OS can be diluted by cross‐over, successive lines of therapy after progression, and non‐cancer‐related death, therefore usually larger samples are required in order to detect OS differences across treatment arms in clinical trials. Moreover, evaluation of OS usually requires a long time to follow‐up. Thus, under the circumstances of rapid development and urgent demand of novel immunotherapies, appropriate surrogate endpoints such as progression‐free survival (PFS) are expected to be applied to assessing the clinical benefit over a shorter period, thereby accelerating the development and introduction of new regimens and drugs into real‐world clinical practice. PFS has been used as a surrogate endpoint in the trials in lung cancer at both trial level and individual‐patient‐data level.^4^, ^5^, ^6^, ^7^, ^8^

Assessment of treatment effect on PFS is based on the determination of response or progression. However, criteria for evaluation of response vary greatly. The World Health Organization (WHO) criteria published in 1979 assess the patient as showing complete response, partial response, stable disease, or progressive disease according to two dimensions, namely, changes in size and number of lesions.^9^ The Response Evaluation Criteria in Solid Tumors (RECIST) specifications, published in 2000, presented measures instead along a single dimension and refined some other details.^10^ In 2009, Response Evaluation Criteria in Solid Tumors version 1.1 (RECIST v1.1) further updated the assessment of tumor burden and lymph nodes, and the confirmation of response based on new clinical evidence.^11^ These are the most frequently used conventional response evaluation criteria in chemotherapy trials. However, the pattern of response to immunotherapy differs from that of response to conventional chemotherapy. Immunotherapy usually takes a longer lag time for a suitable response.^12^ Meanwhile, some patients receiving ICIs might experience enlargement of preexisting lesions or presence of new lesions during the initial phase of treatment due to transient immune cell infiltration and accumulation of cancer cell debris, which is known as pseudoprogression.^13^ The response rate of immunotherapies will be underestimated if assessed per conventional criteria.^14^ Thus, the new response evaluation criteria designed for immunotherapies are warranted to capture actual progression and identify real efficacy in patients receiving ICIs. In 2009, the immune‐related response criteria (irRC) was proposed based on the WHO criteria.^12^ The key point of irRC is ‘wait‐and‐see’. Considering the phenomena of delayed response and pseudoprogression in ICI therapies, immunotherapy will not cease right after the advent of progression assessed per conventional criteria, and assessment of progressive disease is required to be confirmed with a repeated scan at least 4 weeks later. In 2014 and 2017 respectively, two new immune‐related response evaluation criteria, immune‐related Response Evaluation Criteria in Solid Tumors (irRECIST) and iRECIST, were published.^15^, ^16^ Although more details have been refined in these new criteria, the key idea of wait‐and‐see has not changed.

These immune‐related response evaluation criteria were designed based on the atypical pattern of response in patients receiving ICIs, but whether they perform better than conventional response evaluation criteria in assessment of efficacy or clinical benefit in trials of ICIs in lung cancer has not previously been validated at trial level. To assess the survival benefit of an intervention based on the treatment effect on surrogate endpoints, there should be a strong and robust correlation between surrogate endpoints and OS. Thus, this systematic review and meta‐analysis compares the trial‐level correlation between OS and PFS assessed per conventional or immune‐related response evaluation criteria to determine which is the better surrogate endpoint for OS in trials of ICIs in lung cancer.

## 2 MATERIAL AND METHODS

The protocol for this systematic review and meta‐analysis was registered at the PROSPERO International Prospective Register of Systematic Reviews (registration number: CRD42020199492 [Centre for Reviews and Dissemination, University of York, York, United Kingdom]). The methods and reporting of this systematic review and meta‐analysis followed the Preferred Reporting Items for Systematic Reviews and Meta‐analyses (PRISMA) guidelines.

### 2.1 Eligibility criteria

The eligible studies met the following PICOS (participants, interventions, comparisons, outcomes, and study design) criteria. (1) The participants were patients with primary lung cancer (including NSCLC and SCLC). (2) At least one arm of the trial was treated with regimens including ICIs such as the anti‐CTLA‐4 inhibitors and anti‐PD‐1/PD‐L1 inhibitors. (3) The comparisons were not restricted. (4) At least one arm investigating ICIs of the study reported both median PFS and median OS. (5) The study type was limited to prospective clinical trials. The language was not restricted.

### 2.2 Search strategy and study selection

We searched PubMed, EMBASE, and The Cochrane Library for all eligible clinical trials from inception to 4 July 2020. Search terms included ‘lung cancer’, ‘nivolumab’, ‘cemiplimab’, ‘avelumab’, ‘atezolizumab’, ‘durvalumab’, ‘PD‐1’, ‘PD‐L1’, and ‘CTLA‐4’. Duplicate publications were excluded. For multiple publications or results from a single trial in the same patient population, only the latest publication or result was included. Pooled analyses from more than one trial were also excluded. Two investigators (Guang‐Li Zhu and Kai‐Bin Yang) screened the titles and abstracts for potentially eligible studies and then screened the full text of these studies to select fully eligible studies independently. Conference abstracts providing sufficient information were also included. Disagreements between investigators were resolved by consensus or referring to a third investigator (Liang Peng).

### 2.3 Data extraction

Two independent investigators (Guang‐Li Zhu and Kai‐Bin Yang) extracted the following data from eligible studies: clinical trial registration number, any other name of the trial, phase of clinical trial, type of lung cancer, stage of lung cancer, enrollment period, median follow‐up time, number of arms, intervention in each arm, dose of ICIs, intention‐to‐treat sample size of each arm, hazard ratios (HR) for PFS or OS, median OS, median PFS, and criteria for evaluation of response. Missing information could be retrieved from registers such as clinicaltrial.gov according to clinical trial registration number when available.

Disagreements between the two investigators were resolved by consensus or referring to a third investigator (Liang Peng).

### 2.4 Outcome of interest

Clinical outcomes analyzed were OS and PFS. OS was defined as the time from randomization or initiation of treatment until death from any cause. PFS was defined as the time from randomization or initiation of treatment to first progression (locoregional or distant) or death from any cause. According to different response evaluation criteria, the PFS could be denoted as cPFS (assessed per conventional response evaluation criteria) or iPFS (assessed per immune‐related response evaluation criteria). For each comparison between an ICI arm and another arm, the HR for OS and the HR for cPFS/iPFS were paired. For each arm investigating ICIs, the median OS and median cPFS/iPFS were paired.

### 2.5 Statistical analysis

We performed the analysis at the trial or arm level, without individual patient‐level data incorporated. Analysis of trial‐level correlation between OS and PFS included only the treatment arms investigating ICIs. We applied the weighted linear regression model to quantify the trial‐level correlation between the HR of OS and iPFS/cPFS after logarithmic transformation. Missing HRs of OS and iPFS/cPFS were not imputed. Points were weighted by the intention‐to‐treat sample size. We also calculated the surrogate threshold effect (STE) for both criteria. STE is the minimal treatment effect on the surrogate endpoint explaining a nonzero effect on the true endpoint, which is yielded by intersecting the upper prediction limit curve and the horizontal line where HR_OS_ = 1 (zero effect). Besides, we also applied the linear regression model weighted by sample size to quantify the trial‐level correlation between median OS and median iPFS/cPFS. Furthermore, considering the heterogeneity across different phases of trials and different types of lung cancer, ICI and treatment modalities, we performed several sensitivity analyses that stratified the treatment arms by (1) type of lung cancer (SCLC, NSCLC); (2) phase of clinical trials (phase 1 or 1b trials, phase 2 trials); (3) types of ICI (anti‐PD1 or PD‐L1, anti‐CTLA‐4, dual ICI); (4) treatment modalities (ICI alone, ICI + chemotherapy). Only the groups including more than three studies will be included in the stratified analysis. We calculated the weighted coefficient of determination of linear regression (*R*_WLS_^2^) to quantify the variation of OS explained by the iPFS/cPFS. We assessed the strength of correlation as excellent (*R*_WLS_^2^ > 0.90), very good (0.75 < *R*_WLS_^2^ ≤ 0.90), good (0.50 < *R*_WLS_^2^ ≤ 0.75), moderate (0.25 < *R*_WLS_^2^ ≤ 0.50), and poor (*R*_WLS_^2^ ≤ 0.25). Meanwhile, to ensure the robustness of regression and correlation, we applied leave‐one‐out cross validation to each weighted linear regression model and calculated the R^2^ of leave‐one‐out cross validation (*R*_LOO_^2^), root mean squared error, and mean absolute error. Finally, the possibility of publication bias was assessed by visual estimate of the funnel plot and Egger's test when at least 10 trials were pooled.

All statistical analyses were performed by R software (version 3.6.2).

## 3 RESULTS

### 3.1 Selection of studies

**FIGURE 1:**
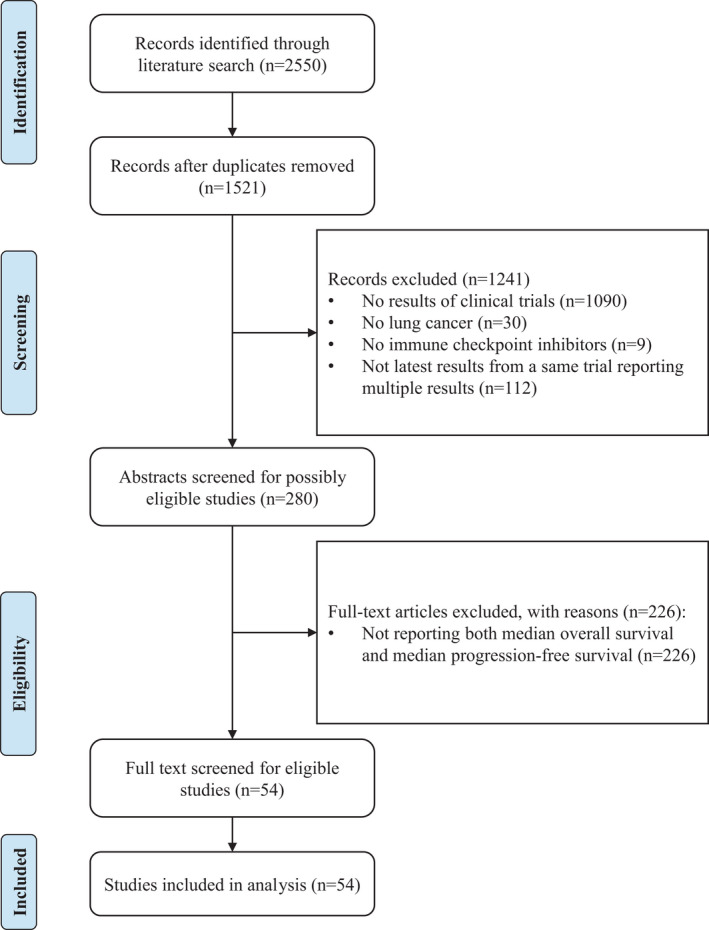
Flowchart of the study selection process

After excluding duplicates, we initially identified a total of 1521 records from PubMed, EMBASE, and The Cochrane Library. After screening the abstracts and titles, we excluded 1241 records, of which 1090 were not results of clinical trials, 30 did not report data on lung cancer, nine did not investigate ICIs, and 112 were not the latest of multiple publications all based upon the same trials. We conducted full‐text review for the remaining 280 potentially eligible studies, among which 226 did not report both median OS and median PFS. Finally, a total of 54 eligible studies^17^, ^18^, ^19^, ^20^, ^21^, ^22^, ^23^, ^24^, ^25^, ^26^, ^27^, ^28^, ^29^, ^30^, ^31^, ^32^, ^33^, ^34^, ^35^, ^36^, ^37^, ^38^, ^39^, ^40^, ^41^, ^42^, ^43^, ^44^, ^45^, ^46^, ^47^, ^48^, ^49^, ^50^, ^51^, ^52^, ^53^, ^54^, ^55^, ^56^, ^57^, ^58^, ^59^, ^60^, ^61^, ^62^, ^63^, ^64^, ^65^, ^66^, ^67^, ^68^ were included for analysis (Figure 1).

### 3.2 Study characteristics

An overview of the included studies is presented in Table S1. A total of 78 arms from all 54 studies, including 13,438 patients, investigated regimens containing ICIs, among which four arms were evaluated per both irRC and modified WHO criteria, one arm was evaluated per irRC and RECIST v1.1, six arms were evaluated per irRC, and one arm was evaluated per irRECIST. For the remaining 66 arms, two arms were evaluated per modified WHO criteria, and 64 arms per RECIST v1.1.

### 3.3 Trial‐level correlation between treatment effect (HR) on OS and PFS assessed per different response evaluation criteria

Table 1, ^2^, ^17^, ^18^, ^19^, ^20^, ^21^, ^22^, ^23^, ^24^, ^25^, ^26^, ^27^, ^28^, ^29^, ^30^, ^31^, ^32^, ^33^, ^34^, ^35^, ^36^, ^37^, ^38^, ^39^, ^40^, ^41^, ^42^, ^43^, ^44^, ^45^, ^46^, ^47^, ^48^, ^49^, ^50^, ^51^, ^52^, ^53^, ^54^, ^55^, ^56^, ^57^, ^58^, ^59^, ^60^, ^61^, ^62^, ^63^, ^64^, ^65^, ^66^, ^67^, ^68^ demonstrates the extracted information about OS and PFS. From the 54 included studies, we retrieved 36 pairs of HR for OS and PFS (30 per conventional response evaluation criteria only, two per irRC only, and four per both criteria).

**TABLE 1:** Progression‐free survival and overall survival from the arms included for analysis

Trials	Regime	Response evaluation criteria	Sample size	Median OS (months)	Median PFS (months)	Hazard ratio of overall survival	Hazard ratio of progression‐free survival
Gadgeel et al. (2020)^17^	Pem + chemo	RECIST v1.1	410	22.0 (19.5–25.2)	9.0 (8.1–9.9)	0.56 (0.45–0.70)	0.48 (0.40–0.58)
Mok et al. (2019)^18^	Pem	RECIST v1.1	637	20.0 (15.4–24.9)	7.1 (5.9–9)	NA	NA
Ott et al. (2017)^19^	Pem	RECIST v1.1	24	9.7 (4.1‐NA)	1.9 (1.7–5.9)	NA	NA
Nishio et al. (2019)^20^	Pem	RECIST v1.1	38	19.2 (8–26.7)	3.9 (2–6.2)	NA	NA
Gubens et al. (2019)^21^	Pem + Ipi	RECIST v1.1	51	10.9 (6.1–23.7)	4.1 (1.4–5.8)	NA	NA
Herbst et al. (2016)^22^	Pem	RECIST v1.1	344	10.4 (9.4–11.9)	3.9 (3.1–4.1)	0.71 (0.58–0.88)	0.88 (0.74–1.05)
	Pem		346	12.7 (10.0–17.3)	4.0 (2.7–4.3)	0.61 (0.49–0.75)	0.79 (0.66–0.94)
Leighl et al. (2019)^23^	Pem (treatment naïve)	RECIST v1.1	101	22.3 (17.1–31.5)	10.3 (8.3–14.7)	NA	NA
	Pem (previously treated)		449	10.5 (8.6–13.2)	4.2 (3.3–4.8)	NA	NA
Reck et al. (2019)^24^	Ate + chemo2	RECIST v1.1	402	21.4 (13.8‐NA)	6.9 (5.7–8.2)	0.93 (0.51–1.68)	1.14 (0.73–1.78)
Horn et al. (2018)^2^	Ate + chemo	RECIST v1.1	201	12.3 (10.8–15.9)	5.2 (4.4–5.6)	0.70 (0.54–0.91)	0.77 (0.62–0.96)
Barlesi et al. (2018)^25^	Ate + chemo	RECIST v1.1	292	18.1 (13‐NA)	7.6 (6.6–8.5)	0.81 (0.64–1.03)	0.60 (0.50–0.72)
Jotte et al. (2020)^26^	Ate + chemo2	RECIST v1.1	338	14.2	6.3	0.88 (0.73–1.05)	0.71 (0.60–0.85)
National Library of Medicine (U.S.). (30 September 2015 – 1 October 2018)^27^	Nivo + Ipi	RECIST v1.1	279	9.2 (8.2–10.3)	1.7 (1.5–2.6)	0.92 (0.75–1.12)	0.72 (0.60–0.87)
	Nivo		280	10.4 (9.5–12.1)	1.9 (1.6–2.6)	0.84 (0.69–1.02)	0.67 (0.56–0.81)
National Library of Medicine (U.S.). (28 August 2015 – 17 August 2018)^28^	Nivo	RECIST v1.1	284	7.5 (5.6–9.2)	1.5 (1.4–1.5)	0.86 (0.72–1.04)	1.41 (1.18–1.69)
Peters et al. (2019)^29^	Nivo + Ipi	RECIST v1.1	396	17.1 (15.0–20.1)	5.1 (4.1–6.3)	0.79 (0.65–0.96)	0.82 (0.69–0.97)
	Nivo		396	15.7 (13.3–18.1)	4.2 (3–5.3)	0.88 (0.75–1.04)	0.99 (0.84–1.17)
National Library of Medicine (U.S.). (10 December 2015 – 15 September 2017)^30^	Nivo	RECIST v1.1	338	11.9 (10.4–14.0)	2.8 (2.4–3.4)	0.68 (0.52–0.9)	0.77 (0.62–0.95)
Antonia et al. (2016)^31^	Nivo	RECIST v1.1	98	4.4 (3.0–9.3)	1.4 (1.4–1.9)	NA	NA
	Niv + Ipi	RECIST v1.1	61	7.7 (3.6–18.0)	2.6 (1.4–4.1)	NA	NA
	Nivo + Ipi	RECIST v1.1	54	6.0 (3.6–11.0)	1.4 (1.3–2.2)	NA	NA
Carbone et al. (2017)^32^	Nivo	RECIST v1.1	271	4.2 (3.0–5.6)	4.2 (3.1–5.5)	1.15 (0.91–1.45)	1.17 (0.95–1.43)
Horn et al. (2017)^33^	Nivo	RECIST v1.1	131	9.2 (7.1–12.6)	3.5 (2.1–5.1)	NA	NA
Horn et al. (2017)^33^	Nivo	RECIST v1.1	287	12.2 (9.7–15.1)	2.3 (2.2–3.4)	NA	NA
Gettinger et al. (2015)^34^	Nivo	RECIST v1.1	129	9.9 (7.8–12.4)	2.3 (1.8–3.7)	NA	NA
Peters et al. (2017)^35^	Ate (no prior chemo)	RECIST v1.1	142	23.5 (18.1‐NA)	5.4 (3.0–6.9)	NA	NA
	Ate (one prior chemo)		271	15.5 (12.3–19.3)	2.8 (1.5–3.9)	NA	NA
	Ate (at least two prior chemo)		254	13.2 (10.7–17.5)	2.8 (2.7–3.0)	NA	NA
Rizvi et al. (2020)^36^	Dur	RECIST v1.1	374	16.3 (12.2–20.8)	4.7 (3.1–6.3)	0.76 (0.56–1.02)	0.87 (0.59–1.29)
	Dur + Tre		372	11.9 (9.0–17.7)	3.9 (2.8–5.0)	0.85 (0.61–1.17)	1.05 (0.72–1.53)
Planchard et al. (2020)^37^	Dur	RECIST v1.1	62	11.7 (8.2–17.4)	3.8 (1.9–5.6)	0.63 (0.42–0.93)	0.71 (0.49–1.04)
	Dur + Tre		174	11.5 (8.7–14.1)	3.5 (2.3–4.6)	0.8 (0.61–1.05)	0.77 (0.59–1.01)
Wrangle et al. (2018)^38^	Nivo + Alt‐803	RECIST v1.1	23	17.4 (9–NA)	9.4 (3‐NA)	NA	NA
Fehrenbacher et al. (2018)^39^	Ate	RECIST v1.1	425	13.8 (11.8–15.7)	2.8 (2.6–3)	0.75 (0.64–0.89)	0.93 (0.8–1.08)
	Ate		613	13.3 (11.3–14.9)	2.7 (2.4–2.9)	0.8 (0.7–0.92)	0.96 (0.85–1.08)
Barlesi et al. (2018)^40^	Ave	RECIST v1.1	396	10.5 (9.2–12.9)	2.8 (2.7–3.5)	0.9 (0.76–1.07)	1.17 (0.98–1.41)
Kanda et al. (2020)^41^	Nivo + chemo1	RECIST v1.1	6	13.2 (11–55.4)	6.3 (0.7–47.8)	NA	NA
	Nivo + chemo2		6	28.5 (14.6–66.2)	11.8 (1.4–65.1)	NA	NA
	Nivo + chemo4		6	12.5 (9.8–16.9)	3.2 (1.9–10.9)	NA	NA
Chen et al. (2020)^42^	Nivo	RECIST v1.1	53	11.5 (6.0–13.7)	1.4 (1.3–2.6)	NA	NA
West et al. (2019)^43^	Ate + chemo	RECIST v1.1	483	18.6 (16.0–21.2)	7 (6.2–7.1)	0.79 (0.64–0.98)	0.64 (0.54–0.77)
Govindan et al. (2017)^44^	Ipi + chemo	mWHO	388	13.4 (11.8–14.8)	5.6 (5.4–5.9)	0.91 (0.77–1.07)	0.87 (0.75–1.01)
Theelen et al. (2019)^45^	Pem + RT	RECIST v1.1	38	15.9 (7.1‐NA)	6.6 (4.0–14.6)	NA	NA
	Pem		40	7.6 (6.0–13.9)	1.9 (1.7–6.9)	1.52 (0.85–2.72)	1.41 (0.85–2.36)
Owonikoko et al. (2019)^46^	Tre/Dur	RECIST v1.1	8	2.6	2.1	NA	NA
	Tre/Dur + SBRT		7	5.7	3.3	1.50 (0.45–4.99)	2.44 (0.75–7.93)
Paz‐Ares et al. (2019)^47^	Dur + chemo	RECIST v1.1	268	13 (11.5–14.8)	5.1 (4.7–6.2)	0.73 (0.59–0.91)	NA
Pujol et al. (2019)^48^	Ate	RECIST v1.1	49	9.5 (3.2–14.4)	1.4 (1.2–1.5)	0.84 (0.45–1.58)	2.26 (1.3–3.93)
Gettinger et al. (2016)^49^	Nivo	RECIST v1.1	52	19.4 (0.5–35.8)	3.6 (0.1–28.0)	NA	NA
National Library of Medicine (U.S.) (13 December 2011 – 19 March 2015)^50^	Ipi + chemo	mWHO	566	10.2 (9.6–10.8)	4.6 (4.5–5.0)	0.96 (0.84–1.10)	0.85 (0.75–0.97)
National Library of Medicine (U.S.) (6 August 2013 – 19 November 2015)^51^	Ate	RECIST v1.1	144	12.6 (9.7–16)	2.7 (2–4.1)	0.69 (0.52–0.92)	0.92 (0.71–1.2)
Goldman et al. (2019)^52^	Nivo (concurrent)	RECIST v1.1	22	29.3 (9.1–8.5)	4.1 (1.3‐NA)	NA	NA
	Nivo (delayed)		10	8.2 (2.2‐NA)	10.5 (4.9–28.4)	NA	NA
Bazhenova et al. (2019)^53^	Nivo + Ipi	RECIST v1.1	125	10.0 (8–12.8)	3.8 (2.3–4.2)	0.97 (0.71–1.31)	0.84 (0.64–1.09)
	Nivo		127	11.0 (8.2–13.5)	2.9 (1.8–3.9)	NA	NA
Cho et al. (2019)^54^	Mk‐1308 + Pem	RECIST v1.1	40	11.0 (5.9–2.0)	1.9 (3.9‐NA)	NA	NA
Paz‐Ares et al. (2018)^55^	Pem + chemo	RECIST v1.1	278	15.9 (13.2‐NA)	6.4 (6.2–8.3)	0.64 (0.49–0.85)	0.56 (0.45–0.7)
Okuma et al. (2018)^56^	Nivo	RECIST v1.1	33	3.8 (2.4–16.3)	1.5 (1.0–2.7)	NA	NA
Lee et al. (2018)^57^	Nivo	RECIST v1.1	100	13.9 (10.8–18.5)	2.8 (1.4–5.7)	NA	NA
Liu et al. (2018)^58^	Ate + chemo1	RECIST v1.1	25	12.9 (8.8–21.3)	7.1 (4.2–8.3)	NA	NA
	Ate + chemo2		25	18.9 (9.9–27.4)	8.4 (4.7–11)	NA	NA
	Ate + chemo3		26	17 (12.7‐NA)	5.7 (4.4–14.8)	NA	NA
Socinski et al. (2019)^59^	Ate	RECIST v1.1	152	14.8 (12.7–21.3)	4.1 (2.8–4.9)	NA	NA
Hida et al. (2017)^60^	Nivo	RECIST v1.1	35	16.3 (12.4–25.4)	4.2 (1.4–7.1)	NA	NA
Sequist et al. (2016)^61^	Ate	RECIST v1.1	17	5.9 (4.3–20.1)	1.5 (1.2–2.7)	NA	NA
Shaverdian et al. (2017)^62^	Pem + RT	irRC	42	10.7 (6.5–18.9)	4.4 (2.1–8.6)	0.58 (0.36–0.94)	0.56 (0.34–0.91)
	Pem		55	5.3 (2.7–7.7)	2.1 (1.6–2.3)	NA	NA
	Pem + extracranial RT		38	11.6 (6.5–20.5)	6.3 (2.1–10.4)	0.59 (0.36–0.94)	0.50 (0.30–0.84)
	Pem		59	5.3 (3–8.5)	2.0 (1.8–2.1)	NA	NA
Lynch et al. (2012)^63^	Ipi + concurrent chemo	irRC	70	11.0 (8.4–12.8)	5.5 (4.2–6.7)	0.96 (0.63–1.48)	0.78 (0.53–1.13)
	Ipi + phased chemo		68	11.6 (9.3–14.4)	5.7 (4.8–7.8)	0.75 (0.48–1.18)	0.69 (0.47–1.01)
	Ipi + concurrent chemo	mWHO	70	11.0 (8.4–12.8)	4.1 (2.8–5.3)	0.96 (0.63–1.48)	0.88 (0.61–1.27)
	Ipi + phased chemo		68	11.6 (9.3–14.4)	5.1 (4.2–5.7)	0.75 (0.48–1.18)	0.69 (0.48–1.00)
Reck et al. (2013)^64^	Ipi + concurrent chemo	irRC	43	9.1 (8.6–11.7)	5.7 (5.2–6.9)	0.95 (0.59–1.54)	0.75 (0.48–1.19)
	Ipi + phased chemo		42	12.9 (7.9–16.5)	6.4 (5.3–7.9)	0.75 (0.46–1.23)	0.64 (0.40–1.02)
	Ipi + concurrent chemo	mWHO	43	9.1 (8.6–11.7)	3.9 (2.9–5.9)	0.95 (0.59–1.54)	0.93 (0.59–1.48)
	Ipi + phased chemo		42	12.9 (7.9–16.5)	5.2 (4.1–6.6)	0.75 (0.46–1.23)	0.93 (0.59–1.45)
Arriola et al. (2016)^65^	Ipi + chemo	irRC	53	17.0 (7.9–24.3)	7.3 (5.5–8.8)	NA	NA
Gadgeel et al. (2017)^66^	Pem	irRC	45	9.2 (6.1–15.2)	4.7 (1.8–6.7)	NA	NA
Mattes et al. (2019)^67^	ICI + RT	irRECIST	34	12.0	6.1	NA	NA
Chiang et al. (2020)^68^	Ate	irRC	17	5.9 (4.3–12.6)	2.9 (1.2–6.1)	NA	NA
		RECIST v1.1	17	5.9 (4.3–12.6)	1.5 (1.2–2.7)	NA	NA

Abbreviations: Ate, atezolizumab; Ave, avelumab; Chemo, chemotherapy; Dur, durvalumab; ICI, immune‐checkpoint inhibitors; Ipi, ipilimumab; NA, not available; Nivo, nivolumab; RT, radiotherapy; Tre, tremelimumab.

**FIGURE 2:**
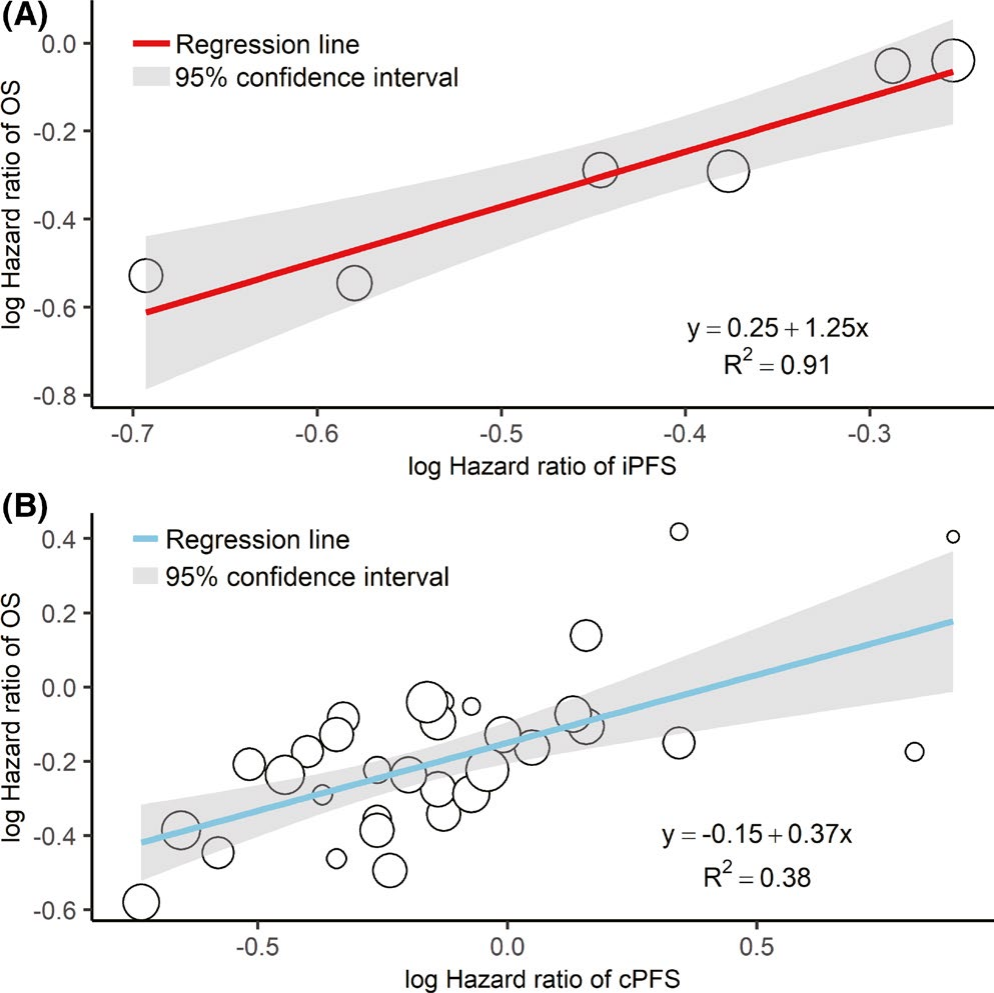
Weighted linear regression between treatment effect (hazard ratio) on OS and iPFS (A) and cPFS (B) after logarithmic transformation. Each circle represents a study, whose size is proportional to the intention‐to‐treat sample size. cPFS, progression‐free survival assessed per conventional response evaluation criteria; iPFS, progression‐free survival assessed per immune‐related response evaluation criteria; OS, overall survival

Table 2 presents the result of weighted linear regression between HR of OS and median iPFS/cPFS for all arms. After logarithmic transformation, the HR of OS had a stronger linear correlation with the HR of iPFS (*R*_WLS_^2^ = 0.91) than with the HR of cPFS (*R*_WLS_^2^ = 0.38; Figure 2). Leave‐one‐out cross validation also confirmed this conclusion. Although the *R*_LOO_^2^ of 0.77 for weighted linear regression between HR of median iPFS and median OS was lower than *R*_WLS_^2^, it still indicates a very good relationship. And the *R*_LOO_^2^ and *R*_WLS_^2^ are the same for the weighted linear regression between HR of median cPFS and median OS, indicating a robust but moderate correlation. Given the limited availability of HR of iPFS, sensitivity analysis was not performed. The STEs for the HR of iPFS and cPFS were 0.75 and 1.21, respectively, which are the maximal HR for observed iPFS and cPFS needed to report possibly significant treatment effect on OS.

**TABLE 2:** Weighted linear correlation between treatment effect (hazard ratio) on overall survival and progression‐free survival

Correlation between	Included arms	Slope	*p*‐value for slope	*R* ^2^ of weighted linear regression	Adjusted *R* ^2^ of eeighted linear regression	*R*_L_^2^ of leave‐one‐out cross validation	Root mean square error	Mean absolute error
Hazard ratio of OS and hazard ratio of cPFS after logarithmic transformation	All arms	0.37	<0.01	0.38	0.36	0.38	0.17	0.13
Hazard ratio of OS and hazard ratio of iPFS after logarithmic transformation	All arms	1.25	<0.01	0.91	0.89	0.77	0.11	0.09

Abbreviations: cPFS, progression‐free survival assessed per conventional response evaluation criteria; iPFS, progression‐free survival assessed per immune‐related response evaluation criteria; OS, overall survival.

### 3.4 Trial‐level correlation between median OS and median iPFS/cPFS

For the ICI arms evaluated by conventional criteria, the mean and standard deviation of median OS and median cPFS were 12.98 ± 5.33 months and 4.30 ± 2.39 months, respectively, whereas the mean and standard deviation of median OS and median iPFS were 10.14 ± 3.44 months and 4.93 ± 1.75 months for the ICI arms evaluated by immune‐related criteria.

**FIGURE 3:**
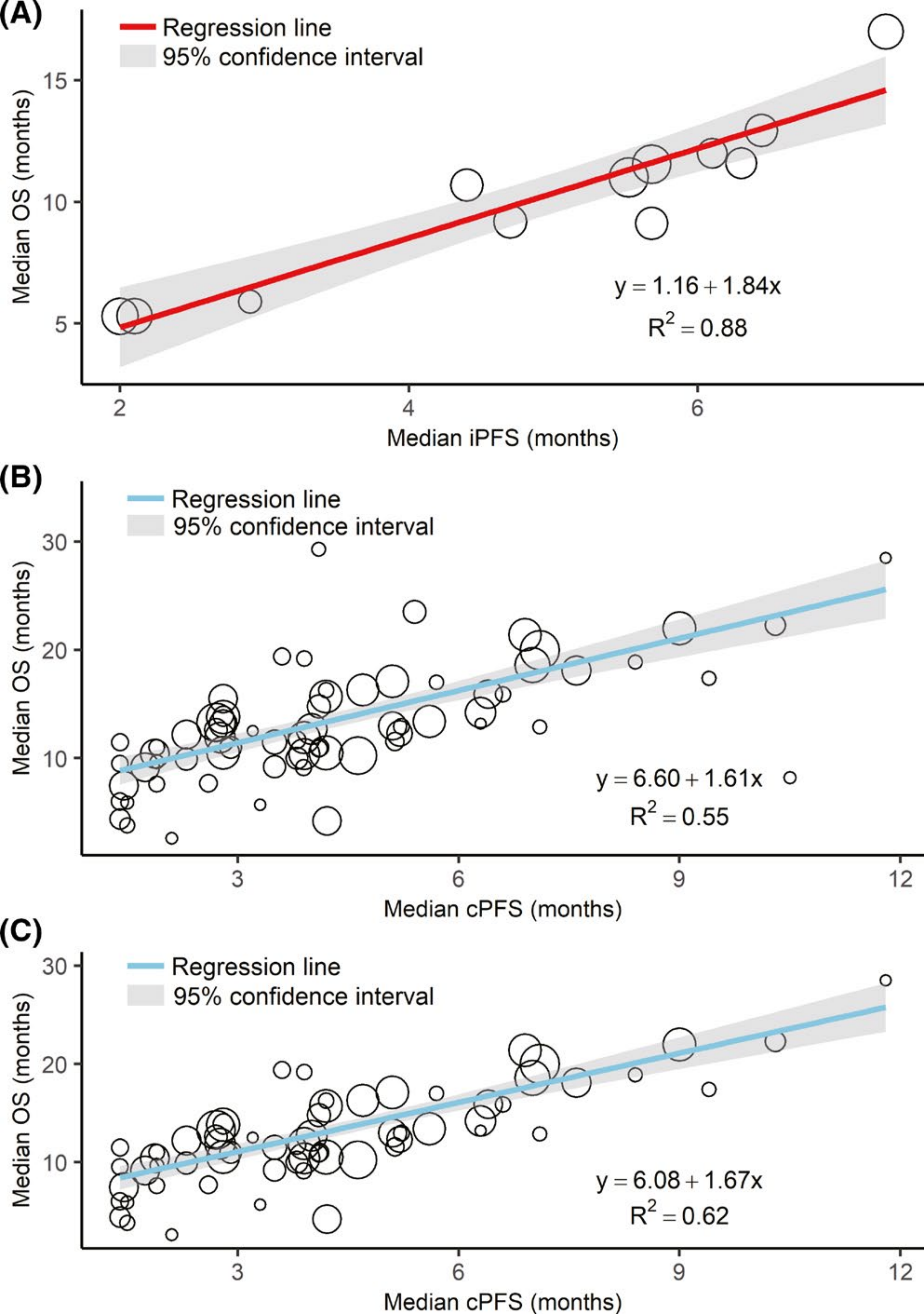
Weighted linear regression between median OS and iPFS (A) and cPFS (B) and cPFS after removal of two outlier studies (C). Each study is represented by a circle whose size is proportional to the intention‐to‐treat sample size. cPFS, progression‐free survival assessed per conventional response evaluation criteria; iPFS, progression‐free survival assessed per immune‐related response evaluation criteria; OS, overall survival

Table 3 demonstrates the result of weighted linear regression between median OS and median iPFS/cPFS for all arms and different subgroups. The correlation between median OS and median iPFS was very good (*R*_WLS_^2^ = 0.88; Figure 3A), while the correlation between median OS and median cPFS was weaker (*R*_WLS_^2^ = 0.55) but still good (Figure 3B). Outliers are data points with studentized residual outside the ±2 range. There are two notable outliers: studies reported by Peters et al.^35^ and Goldman et al.^52^ After we excluded these two studies, there was a slight change in the slope and intercept of the weighted linear regression model but an obvious increase in *R*_WLS_^2^ to 0.62 (Figure 3C). In the leave‐one‐out cross validation, the correlation between median OS and median cPFS (*R*_LOO_^2^ = 0.40), or even the correlation after excluding the two outlier studies (*R*_LOO_^2^ = 0.62), was still weaker than the correlation between median OS and median iPFS (*R*_LOO_^2^ = 0.81).

**TABLE 3:** Weighted linear correlation between overall survival and progression‐free survival

Correlation between	Included arms	Slope of weighted linear regression	*p*‐value for slope	*R* ^2^ of weighted linear regression	Adjusted *R* ^2^ of weighted linear regression	*R*_L_^2^ of leave‐one‐out cross validation	Root mean square error	Mean absolute error
Median OS and median cPFS	Overall							
	All arms	1.61	<0.01	0.55	0.54	0.40	4.17	3.01
	All arms excluding studies by Peters et al.^35^ and Goldman et al.^52^	1.67	<0.01	0.62	0.61	0.62	3.06	2.48
	Type of lung cancer							
	SCLC	1.27	<0.01	0.67	0.65	0.45	2.44	2.01
	NSCLC	1.53	<0.01	0.51	0.50	0.27	4.51	3.18
	NSCLC excluding studies by Peters et al.^35^ and Goldman et al.^52^	1.61	<0.01	0.59	0.58	0.56	3.15	2.56
	Phase of clinical trials							
	Phase 1 arms	1.52	<0.01	0.51	0.48	0.27	5.91	4.04
	Phase 1 arms excluding study by Goldman et al.^52^	2.10	<0.01	0.80	0.76	0.66	3.57	2.77
	Phase 2 arms	1.70	<0.01	0.28	0.25	0.30	4.01	3.48
	Phase 2 arms excluding study by Peters et al.^35^	1.48	<0.01	0.41	0.38	0.39	3.07	2.64
	Type of ICI							
	Anti‐PD‐1/anti‐PD‐L1	1.59	<0.01	0.57	0.57	0.38	4.46	3.10
	Anti‐CTLA‐4	2.78	<0.01	0.80	0.76	0.62	1.11	0.94
	Dual ICI	2.40	<0.01	0.81	0.79	0.28	3.51	3.05
	Treatment modalities							
	ICI alone	1.71	<0.01	0.46	0.44	0.23	4.74	3.32
	ICI + chemotherapy	2.73	<0.01	0.84	0.83	0.79	2.43	1.87
Median OS and median iPFS	All arms	1.84	<0.01	0.88	0.87	0.81	1.43	1.03
	Type of lung cancer							
	SCLC arms	2.62	0.02	0.86	0.81	0.66	2.64	2.26
	NSCLC arms	1.62	<0.01	0.96	0.95	0.93	0.75	0.56
	Phase of clinical trials							
	Phase 1 arms	1.64	<0.01	0.92	0.90	0.81	1.33	0.95
	Phase 2 arms	3.05	<0.01	0.86	0.83	0.62	1.55	1.27
	Type of ICI							
	Anti‐PD‐1/Anti‐PD‐L1	1.58	<0.01	0.93	0.90	0.84	1.12	0.93
	Anti‐CTLA‐4	3.54	0.02	0.88	0.83	0.65	1.59	1.46
	Treatment modalities							
	ICI + chemotherapy	3.54	0.02	0.88	0.83	0.65	1.59	1.46

Abbreviations: cPFS, progression‐free survival assessed per conventional criteria; ICI, immune‐checkpoint inhibitors; NSCLC, non‐small cell lung cancer; OS, overall survival; SCLC, small‐cell lung cancer.

### 3.5 Sensitivity analyses

We first performed sensitivity analysis according to the type of lung cancer. For arms investigating SCLC, the median iPFS (*R*_WLS_^2^ = 0.86) showed a better correlation with median OS than did median cPFS (*R*_WLS_^2^ = 0.67). Similarly, for arms investigating NSCLC, the median iPFS also showed a stronger correlation with median OS (*R*_WLS_^2^ = 0.96) than did median cPFS (*R*_WLS_^2^ = 0.51). Excluding the studies by Peters et al.^35^ and Goldman et al.^52^ improved the correlation between median OS and median cPFS in NSCLC (*R*_WLS_^2^ = 0.59), but it was still much weaker than the correlation between median OS and median iPFS. Leave‐one‐out cross validation also confirmed this conclusion. *R*_LOO_^2^ improved from 0.27 to 0.56 after removal of the studies by Peters et al.^35^ and Goldman et al.,^52^ indicating an increase in robustness of correlation between median OS and median cPFS, but still did not match the excellent correlation between median OS and median iPFS (*R*_LOO_^2^ = 0.93).

Besides, we also performed sensitivity analysis according to the phase of clinical trials. In the trials assessed per conventional criteria, phase 3 trials (44.7%) predominated, followed by phase 2 trials (23.4%) and phase 1 or 1b trials (23.4%), whereas in the trials assessed per immune‐related criteria, phase 2 trials (58.3%) predominated, followed by phase 1 trials (41.7%). For arms from phase 1 or phase 1b trials, the median iPFS showed a much stronger correlation with median OS (*R*_WLS_^2^ = 0.92) than did the median cPFS (*R*_WLS_^2^ = 0.51). After removal of the outlier study by Goldman et al.,^52^ the *R* ^2^ of weighted linear regression between cPFS and OS (*R*_WLS_^2^ = 0.76) increased, and the *R*_LOO_^2^ also improved significantly to 0.66 in leave‐one‐out validation. Similarly for arms from phase 2 trials, the median iPFS still showed a better correlation with median OS (*R*_WLS_^2^ = 0.86) than did median cPFS (*R*_WLS_^2^ = 0.28). And the removal of the outlier study by Peters et al.^35^ mildly improved the *R* ^2^ of weighted linear regression to *R*_WLS_^2^ = 0.41 and of leave‐one‐out cross validation to *R*_LOO_^2^ = 0.39.

Finally, we performed sensitivity analysis based on the types of ICI and treatment modalities, respectively. Regarding the types of ICI, concerning that only 1 trial used anti‐PD‐L1 antibody among the trials assessed per immune‐related criteria, we combined anti‐PD1 and anti‐PD‐L1 antibody as one group. The correlation between median OS and median iPFS were very good and excellent in anti‐CTLA‐4 group (*R*_WLS_^2^ = 0.93) and anti‐PD1/anti‐PD‐L1 group (*R*_WLS_^2^ = 0.88), respectively, while a very good and good correlation were detected between median OS and median cPFS in anti‐CTLA‐4 group (*R*_WLS_^2^ = 0.80) and anti‐PD1/anti‐PD‐L1 group (*R*_WLS_^2^ = 0.57), respectively. As for the types of treatment modalities, limited by the number of trials, the stratified analysis could not be performed in ICI alone group for iPFS and ICI + radiotherapy group for both iPFS and cPFS. We detected a moderate and very good correlation between OS and cPFS in ICI alone group (*R*_WLS_^2^ = 0.46) and ICI + chemotherapy group (*R*_WLS_^2^ = 0.84), respectively. However, the correlation between OS and iPFS in ICI + chemotherapy (*R*_WLS_^2^ = 0.86) group was stronger. In addition, correlation between median OS and median iPFS (*R*_LOO_^2^ ranging from 0.65 to 0.84) were also more robust than median OS and median cPFS (*R*_LOO_^2^ ranging from 0.28 to 0.79) as demonstrated in the leave‐one‐out cross validation.

### 3.6 Assessment of publication bias

The funnel plot is highly symmetric, and Egger's test shows no evidence of publication bias in the arms reporting HR of OS (*p* = 0.66) and PFS (*p* = 0.64) assessed per conventional response evaluation criteria (Figure S1). Fewer than 10 arms reported HR of OS and PFS assessed per immune‐related response evaluation criteria, so their publication bias was not evaluated.

## 4 DISCUSSION

The first ICI used to treat advanced NSCLC, nivolumab, was approved in 2015. In 2016, pembrolizumab was approved as a first‐line treatment option for metastatic NSCLC. Great breakthroughs by the emerging ICIs in prolonging the survival of patients with advanced lung cancer have elicited a rapidly increased number of trials of ICIs in lung cancer. An appropriate surrogate endpoint for OS to predict clinical benefit at an early phase of trials and accelerate patients’ access to new ICIs is therefore urgently needed. In this study, we aimed at comparing the correlation between OS and PFS assessed per immune‐related and conventional response evaluation criteria in lung cancer patients receiving ICIs at trial level.

The trial‐level correlation between the HR of OS and cPFS was moderate (*R*_WLS_^2^ = 0.36). The trial‐level correlation between OS and cPFS was worse than in previously reported studies, which included only trials of conventional chemotherapy.^4^, ^5^, ^6^, ^8^ Similarly, there is a good or very good trial‐level correlation between median OS and median cPFS (*R*_WLS_^2^ ranging from 0.51 to 0.80) for all arms and subgroups except phase 2 trials or trials using ICI alone, which only exhibited moderate correlation even after the removal of the outlier studies. However, in the leave‐one‐out cross validation for all arms and subgroups, we only detected a *R*_LOO_^2^ greater than 0.5 in three subgroups, indicating a moderate power of prediction. The atypical pattern of response and various regimens of ICIs with or without conventional cytotoxic drugs might lower the predictive value of cPFS as a surrogate marker under these less restrictive circumstances. Thus, the above evidence provides only moderate support for considering cPFS as an appropriate surrogate endpoint for OS in trials of ICIs in lung cancer.

Conversely, the trial‐level correlation between the HR of OS and iPFS was excellent (*R*_WLS_^2^ = 0.91), which was validated by the leave‐one‐out cross validation (*R*_LOO_^2^ = 0.77). STE was 0.75 for iPFS, indicating that with an HR of iPFS lower than 0.75 we could predict a statistically significant HR of OS. Moreover, the considerably higher STE of 1.21 for cPFS means that even patients with worse cPFS under immunotherapy than control treatment (PFS HR >1), can derive statistically significant OS benefit with immunotherapy compared to control treatment, indicating the underestimation of the OS benefit of immunotherapy with the cPFS evaluation per conventional criteria. Moreover, we also demonstrated a very good trial‐level correlation between the median OS and median iPFS for all arms (*R*_WLS_^2^ = 0.88). And the *R*_LOO_^2^ was greater than 0.60 for all arms and subgroups in the leave‐one‐out cross validation. The strong and robust trial‐level correlation suggest that median iPFS and HR of iPFS are appropriate surrogate endpoints in trials of ICIs in lung cancer. Another meta‐analysis including 14 randomized controlled trials with patients across five types of cancers reported a slightly stronger but still moderate trial‐level correlation between iPFS and OS (*R* ^2^ = 0.277) compared with the correlation between cPFS and OS (*R* ^2^ = 0.260), which might be associated with heterogeneity of patterns of survival in patients across different types of cancer.^69^

The importance of adopting a response evaluation criteria adaptive to the unique pattern of response to immune‐checkpoint inhibitors has not received adequate attention. Although it has been over 10 years since the publication of the irRC, RECIST v1.1 is still the most frequently used set of criteria for response evaluation in clinical trials of ICIs. The immune‐related response evaluation criteria were mainly used in early phases of trials, and no result of phase 3 trials evaluated per immune‐related response evaluation criteria has yet been identified. There were several reasons why immune‐related response criteria were still not widely used in ICI trials. First, prospective randomized trials comparing conventional and immune‐related response criteria have yet to be conducted, so there was no confirmatory evidence of the superiority of immune‐related response criteria over conventional criteria. In fact, the irRC guidelines did not claim its superiority in the response evaluation in ICI trials, but only recommended prospective validation of the new criteria in the future trials.^12^ RECIST criteria are still the most widely used and recognized criteria. Second, implementation of trials with immune‐related criteria requires more precautions due to the risk of continuing the treatment after documented progression. Third, the immune‐related response criteria were made partly in response to the findings of atypical patterns of response to ICI. However, some oncologists think that the rates of pseudoprogression, which was reported to be less than 10%,^70^ is insufficient to lead to a significantly difference in the assessment of PFS, while some oncologists thought that PFS in ICI trials would be better assessed with these new criteria. Thus, this study tries to provide an insight into the importance of immune‐related response evaluation criteria in immune‐checkpoint inhibitor trials even with limited availability of studies assessed per immune‐related response evaluation criteria.

The strength of the evidence of this study was mainly restricted by the limited availability of studies assessed per immune‐related response evaluation criteria. The gold standard of surrogate endpoint analysis is the correlation between HR on cPFS or iPFS and HR on OS. However, the lack of phase 3 trials results in a lack of direct comparison of correlation for both types of PFS, which leads to insufficient data on HR. And further analysis, including sensitivity analysis, could not be performed for the correlation between the HR of OS and iPFS. In this study, to further validate the superiority of immune‐related response evaluation criteria over conventional criteria, we additionally analyzed the correlation between medians of cPFS or iPFS and medians of OS to make more confirmatory conclusions. Besides, another concern with the lack of Phase III trials with iPFS in this analysis is that the correlation between OS and PFS could be confounded by trial phase. In this study, the lower correlation and poorer prediction power of cPFS could be due to the broader and more heterogeneous patients enrolled in phase 3 trials, while phase 1 and 2 trials are usually more stringent. However, the strong correlation between median OS and median iPFS persisted in the subgroups of different phases of trials and different types of lung cancer, ICI, and treatment modalities (*R*_WLS_^2^ ranging from 0.86 to 0.96), suggesting that the correlation between median OS and median iPFS might not be influenced by differences in phase of trials and types of lung cancer, ICI, and treatment modalities. Furthermore, a surrogate endpoint for OS would be more useful in phase 3 trials than phase 1 or 2 trials, because it usually takes a much longer time to follow‐up in phase 3 trials.

This research also has other limitations. First, although an appropriate surrogate endpoint should be validated at both trial level and individual‐patient‐data level, we did not incorporate individual‐patient‐data due to the lack of corresponding data. For the design of trials for ICIs in the future, the adoption of immune‐related response evaluation criteria should be considered, and the surrogacy of iPFS should be further validated at individual‐patient‐data level in the future studies. Second, the update in iRECIST has overcome many disadvantages of previous criteria including irRC and irRECIST, but no data on trials assessed per iRECIST are available. Thus, whether the update in iRECIST could improve the correlation between OS and PFS compared with irRC or irRECIST requires further study.

In conclusion, this systematic review and meta‐analysis demonstrates a strong trial‐level correlation between treatment effect (HR) on OS and iPFS. Similarly, a strong and robust trial‐level correlation between median OS and iPFS across different phases of trials and different types of lung cancer, ICI, and treatment modalities in trials of ICIs in lung cancer were also presented. It suggests that iPFS provides valid and robust surrogacy for OS in trials of ICIs in lung cancer. Conversely, the moderate correlation between OS and cPFS provides only modest support for adopting cPFS as surrogate endpoint for OS in trials of ICIs in lung cancer. The conclusion should be further validated at the individual‐patient‐data level and phase 3 trials.

## CONFLICTS OF INTEREST

The authors declare that there is no conflict of interest.

## ETHICAL STATEMENT

Ethics approval was not required for this study.

## Supporting information

Figure S1

Table S1

## Data Availability

The data that support the findings of this study are openly available in Pubmed at https://pubmed.ncbi.nlm.nih.gov/, Embase at https://www.embase.com/, and Cochrane library at https://www.cochranelibrary.com.
