# Supplementary figures and images for: Progression‐free survival assessed per immune‐related or conventional response criteria, which is the better surrogate endpoint for overall survival in trials of immune‐checkpoint inhibitors in lung cancer: A systematic review and meta‐analysis
Source: Cancer Med. 2021 Oct 20;10(23):8272–87. doi: 10.1002/cam4.4347 (PMC8633231; doi:10.1002/cam4.4347)

A

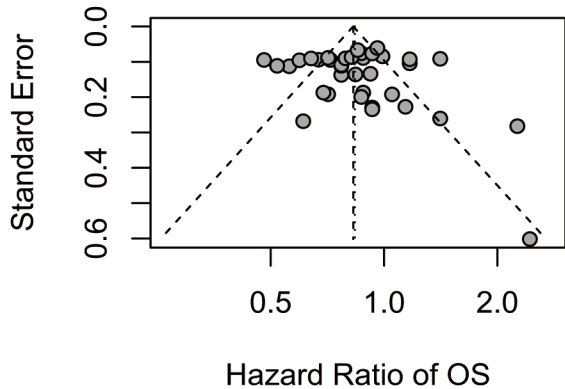

B

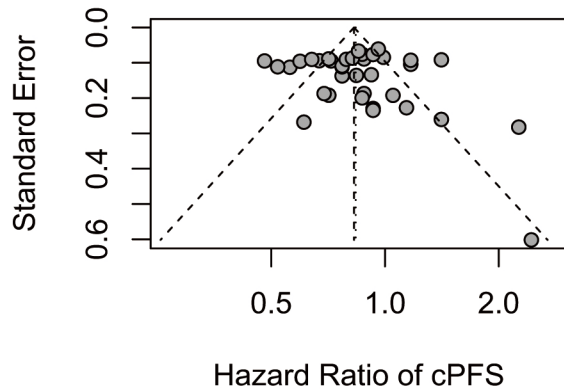

Supplement: Supplementary file 1 — Figure S1 [file CAM4-10-8272-s001.pdf]
