# Supplementary material for: Progression‐free survival assessed per immune‐related or conventional response criteria, which is the better surrogate endpoint for overall survival in trials of immune‐checkpoint inhibitors in lung cancer: A systematic review and meta‐analysis
Source: Cancer Med. 2021 Oct 20;10(23):8272–87. doi: 10.1002/cam4.4347 (PMC8633231; doi:10.1002/cam4.4347)
Supplement: Supplementary file 2 — Table S1 [file CAM4-10-8272-s002.docx]

**Supplementary Table S1.** Description of the Included Studies

| **Trials** | **Registration Number** | **Phase of Trials** | **Type of Lung Cancer** | **Tumor stage** | **Enrollment Period** | **Median Follow-up (months)** | **Arm Number** | **Regime** | **ICI Dose** | **ICI Type** | **Response Evaluation Criteria** | **Sample Size** |
| --- | --- | --- | --- | --- | --- | --- | --- | --- | --- | --- | --- | --- |
| Shirish Gadgeel (2020) ^17^ | NCT02578680  (KEYNOTE-189) | III | NSCLC | IV | 2016-2017 | 23.1 | 2 | Pem + Chemo | 200 mg Q3W | anti-PD-1 | RECIST v1.1 | 410 |
|  |  |  |  |  |  |  |  | Chemo | - |  |  | 206 |
| Tony S. K. Mok (2019) ^18^ | NCT02220894  (KEYNOTE-042) | III | NSCLC | Locally advanced or metastatic | 2014-2017 | 12.8 | 2 | Pem | 200 mg Q3W | anti-PD-1 | RECIST v1.1 | 637 |
|  |  |  |  |  |  |  |  | Chemo | - |  |  | 637 |
| Patrick A. Ott (2017) ^19^ | NCT02054806  (KEYNOTE-028) | Ib | SCLC | Extensive-Stage | 2014-2015 | 9.8 | 1 | Pem | 10 mg/kg Q2W×24 months | anti-PD-1 | RECIST v1.1 | 24 |
| Makoto Nishio (2019) ^20^ | NCT02007070  (KEYNOTE-025) | Ib | NSCLC | Advanced | 2014 | 19.2 | 1 | Pem | 10 mg/kg Q3W | anti-PD-1 | RECIST v1.1 | 38 |
| Matthew A. Gubens (2019) ^21^ | NCT02039674  (KEYNOTE-021) | I/II | NSCLC | IIIB/IV | 2014-2015 | 11.3 | 1 | Pem + Ipi | Pem: 2 mg/kg  Ipi: 1 mg/kg | Pem: anti-PD-1 Ipi: anti-CTLA-4 | RECIST v1.1 | 51 |
| Roy S. Herbst (2016) ^22^ | NCT01905657  (KEYNOTE-010) | II/III | NSCLC | Not restricted | 2013-2015 | 13.1 | 3 | Pem | 2 mg/kg Q3W | anti-PD-1 | RECIST v1.1 | 344 |
|  |  |  |  |  |  |  |  | Pem | 10 mg/kg Q3W | anti-PD-1 |  | 346 |
|  |  |  |  |  |  |  |  | Chemo | - |  |  | 343 |
| Natasha B. Leighl (2019) ^23^ | NCT01295827  (KEYNOTE-001) | I | NSCLC | Locally advanced or metastatic | 2012-2014 | 34.5 | 2 | Pem (Treatment Naïve) | 2 mg/kg or 10 mg/kg Q3W or 10 mg/kg Q2W | anti-PD-1 | RECIST v1.1 | 101 |
|  |  |  |  |  |  |  |  | Pem (Previously Treated) |  |  |  | 449 |
| Martin Reck (2019) ^24^ | NCT02366143  (IMpower150) | III | NSCLC | IV | 2015-2016 | 19.6 | 3 | Ate + Chemo1 | 1200 mg Q3W | anti-PD-L1 | RECIST v1.1 | 400 |
|  |  |  |  |  |  |  |  | Ate + Chemo2 |  |  |  | 402 |
|  |  |  |  |  |  |  |  | Chemo |  |  |  | 400 |
| Leora Horn (2018) ^2^ | NCT02763579  (IMpower133) | I/II | SCLC | Extensive-Stage | 2016-2017 | 13.9 | 2 | Ate + Chemo | 1200 mg Q3W | anti-PD-L1 | RECIST v1.1 | 201 |
|  |  |  |  |  |  |  |  | Chemo | - |  |  | 202 |
| Fabrice Barlesi (2018) ^25^ | NCT02657434  (IMpower132) | III | NSCLC | IV | NA | 14.8 | 2 | Ate + Chemo | 1200 mg Q3W | anti-PD-L1 | RECIST v1.1 | 292 |
|  |  |  |  |  |  |  |  | Chemo | - |  |  | 286 |
| Robert Jotte (2020) ^26^ | NCT02367794  (IMpower131) | III | NSCLC | IV | 2015-2017 | 18.1 | 3 | Ate + Chemo1 | 1200 mg Q3W | anti-PD-L1 | RECIST v1.1 | 343 |
|  |  |  |  |  |  |  |  | Ate + Chemo2 |  |  |  | 338 |
|  |  |  |  |  |  |  |  | Chemo | - |  |  | 340 |
| - ^27^ | NCT02538666  (CheckMate 451) | III | SCLC | Extensive-Stage | NA | NA | 3 | Nivo + Ipi | Nivo: 1 mg/kg Q3W Ipi: 3 mg/kg Q3W | Nivo: anti-PD-1 Ipi: anti-CTLA-4 | RECIST v1.1 | 279 |
|  |  |  |  |  |  |  |  | Nivo | 240 mg Q2W | anti-PD-1 |  | 280 |
|  |  |  |  |  |  |  |  | Placebo | - |  |  | 275 |
| - ^28^ | NCT02481830  (CheckMate331) | III | SCLC | Relapsed | NA | NA | 2 | Nivo | 240 mg Q2W | anti-PD-1 | RECIST v1.1 | 284 |
|  |  |  |  |  |  |  |  | Chemo | - |  |  | 285 |
| Solange Peters (2019) ^29^ | NCT02477826  (CheckMate 227) | III | NSCLC | IV or recurrent | 2015-2016 | NA | 3 | Nivo + Ipi | Nivo: 3 mg/kg Q2W Ipi: 1 mg/kg Q6W | Nivo: anti-PD-1 Ipi: anti-CTLA-4 | RECIST v1.1 | 396 |
|  |  |  |  |  |  |  |  | Nivo | 240 mg Q2W | anti-PD-1 |  | 396 |
|  |  |  |  |  |  |  |  | Chemo | - |  |  | 397 |
| - ^30^ | NCT02613507  (CheckMate 078) | III | NSCLC | IIIB/IV or recurrent | NA | NA | 2 | Nivo | 3 mg/kg Q2W | anti-PD-1 | RECIST v1.1 | 338 |
|  |  |  |  |  |  |  |  | Chemo | - |  |  | 166 |
| Scott J. Antonia (2016) ^31^ | NCT01928394  (CheckMate 032) | I/II | SCLC | Not restricted | 2013-2015 | 28.4 | 3 | Nivo | 3 mg/kg Q2W | anti-PD-1 | RECIST v1.1 | 98 |
|  |  |  |  |  |  |  |  | Niv + Ipi | Nivo: 1 mg/kg Q2W Ipi: 3 mg/kg Q3W | Nivo: anti-PD-1 Ipi: anti-CTLA-4 | RECIST v1.1 | 61 |
|  |  |  |  |  |  |  |  | Nivo + Ipi | Nivo: 3 mg/kg Q2W Ipi: 1 mg/kg Q3W | Nivo: anti-PD-1 Ipi: anti-CTLA-4 | RECIST v1.1 | 54 |
| David P. Carbone (2017) ^32^ | NCT02041533  (CheckMate 026) | III | NSCLC | IV or recurrent | 2014-2015 | 13.5 | 2 | Nivo | 3 mg/kg Q2W | anti-PD-1 | RECIST v1.1 | 271 |
|  |  |  |  |  |  |  |  | Chemo | - |  |  | 270 |
| Leora Horn (2017) ^33^ | NCT01642004  (CheckMate 017) | III | NSCLC | IIIB/IV or recurrent | 2012-2013 | NA | 2 | Nivo | 3 mg/kg Q2W | anti-PD-1 | RECIST v1.1 | 131 |
|  |  |  |  |  |  |  |  | Chemo | - |  |  | 129 |
| Leora Horn (2017) ^33^ | NCT01673867  (CheckMate 057) | III | NSCLC | IIIB/IV or recurrent | 2012-2013 | NA | 2 | Nivo | 3 mg/kg Q2W | anti-PD-1 | RECIST v1.1 | 287 |
|  |  |  |  |  |  |  |  | Chemo | - |  |  | 268 |
| Scott N. Gettinger (2015) ^34^ | NCT00730639 | I | NSCLC | Advanced or recurrent | 2008-2012 | 39.0 | 1 | Nivo | 1, 3, or 10 mg/kg Q2W | anti-PD-1 | RECIST v1.1 | 129 |
| Solange Peters (2017) ^35^ | NCT02031458 | II | NSCLC | IIIB/IV or recurrent | 2014 | 14.6 | 3 | Ate (No Prior Chemo) | 1200 mg Q3W | anti-PD-L1 | RECIST v1.1 | 142 |
|  |  |  |  |  |  |  |  | Ate (One Prior Chemo) |  |  |  | 271 |
|  |  |  |  |  |  |  |  | Ate (At Least Two Prior Chemo) |  |  |  | 254 |
| Naiyer A. Rizvi (2020) ^36^ | NCT02453282  (MYSTIC) | III | NSCLC | IV | 2015-2016 | 30.2 | 3 | Dur | 20 mg/kg Q4W | anti-PD-L1 | RECIST v1.1 | 374 |
|  |  |  |  |  |  |  |  | Dur + Tre | Dur: 20 mg/kg Q4W Tre: 1 mg/kg Q4W | Dur: anti-PD-L1 Tre: anti-CTLA-4 |  | 372 |
|  |  |  |  |  |  |  |  | Chemo | - |  |  | 372 |
| David Planchard (2020) ^37^ | NCT02352948  (ARCTIC) | III | NSCLC | IIIB/ IV | 2015-2016 | 9.1 | 4 | Dur | 10 mg/kg Q2W | anti-PD-L1 | RECIST v1.1 | 62 |
|  |  |  |  |  |  |  |  | Standard Of Care | - |  |  | 64 |
|  |  |  |  |  |  |  |  | Dur + Tre | Dur: 20 mg/kg Q2W Tre: 1 mg/kg Q4W | Dur: anti-PD-L1 Tre: anti-CTLA-4 |  | 174 |
|  |  |  |  |  |  |  |  | Standard Of Care | - |  |  | 118 |
| John M. Wrangle (2018) ^38^ | NCT02523469 | Ib | NSCLC | IIIB/ IV | 2016-2017 | 6.9 | 1 | Nivo + Alt-803 | 3 mg/kg Q2W | anti-PD-1 | RECIST v1.1 | 23 |
| Louis Fehrenbacher (2018) ^39^ | NCT02008227  (OAK) | III | NSCLC | IIIB/ IV or recurrent | 2014 | 28.0 | 4 | Ate | 1200 mg Q3W | anti-PD-L1 | RECIST v1.1 | 425 |
|  |  |  |  |  |  |  |  | Chemo | - |  |  | 425 |
|  |  |  |  |  |  |  |  | Ate | 1200 mg Q3W | anti-PD-L1 |  | 613 |
|  |  |  |  |  |  |  |  | Chemo | - |  |  | 612 |
| Fabrice Barlesi (2018) ^40^ | NCT02395172  (JAVELIN Lung 200) | III | NSCLC | IIIB/ IV or recurrent | 2015-2017 | 18.3 | 2 | Ave | 10 mg/kg Q2W | anti-PD-L1 | RECIST v1.1 | 396 |
|  |  |  |  |  |  |  |  | Chemo | - |  |  | 396 |
| Shintaro Kanda (2020) ^41^ | JapicCTI-132071 | Ib | NSCLC | IIIB/ IV or recurrent | 2013-2014 | NA | 4 | Nivo + Chemo1 | 10 mg/kg Q3W | anti-PD-1 | RECIST v1.1 | 6 |
|  |  |  |  |  |  |  |  | Nivo + Chemo2 |  |  |  | 6 |
|  |  |  |  |  |  |  |  | Nivo + Chemo3 |  |  |  | 6 |
|  |  |  |  |  |  |  |  | Nivo + Chemo4 |  |  |  | 6 |
| Yuh-Min Chen (2020) ^42^ | NCT02582125 | II | NSCLC | IIIB/ IV or recurrent | NA | NA | 1 | Nivo | 3 mg/kg Q2W | anti-PD-1 | RECIST v1.1 | 53 |
| Howard West (2019) ^43^ | NCT02367781  (IMpower130) | III | NSCLC | IV | 2015-2017 | 18.5 | 2 | Ate + Chemo | 1200 mg Q3W | anti-PD-L1 | RECIST v1.1 | 483 |
|  |  |  |  |  |  | 19.2 |  | Chemo | - |  |  | 240 |
| Ramaswamy Govindan (2017) ^44^ | NCT01285609 | III | NSCLC | IV or recurrent | 2011-2015 | 12.5 | 2 | Ipi + Chemo | 10 mg/kg Q3W | anti-CTLA-4 | mWHO | 388 |
|  |  |  |  |  |  | 11.8 |  | Chemo | - |  |  | 361 |
| Willemijn S. M. E. Theelen (2019) ^45^ | NCT02492568 | II | NSCLC | IV | 2015-2018 | 23.6 | 2 | Pem + RT | 200 mg/kg Q3W | anti-PD-1 | RECIST v1.1 | 38 |
|  |  |  |  |  |  |  |  | Pem |  |  |  | 40 |
| Taofeek Kunle Owonikoko (2019) ^46^ | NCT02701400 | II | SCLC | Relapsed | NA | NA | 2 | Tre/Dur | Dur: 75 mg Q4W Tre: 1500 mg Q4W | Dur: anti-PD-L1 Tre: anti-CTLA-4 | RECIST v1.1 | 8 |
|  |  |  | SCLC |  |  |  |  | Tre/Dur + SBRT |  |  |  | 7 |
| Luis Paz-Ares (2019) ^47^ | NCT03043872  (CASPIAN) | III | SCLC | Extensive-Stage | 2017-2018 | 14.2 | 3 | Dur + Chemo | 1500 mg Q3W | anti-PD-L1 | RECIST v1.1 | 268 |
|  |  |  |  |  |  |  |  | Dur + Tre + Chemo | Dur: 1500 mg Q3W Tre: 75 mg Q3W | Dur: anti-PD-L1 Tre: anti-CTLA-4 |  | 268 |
|  |  |  |  |  |  |  |  | Chemo | - |  |  | 269 |
| Jean-Louis Pujol (2019) ^48^ | NCT03059667 | II | SCLC | Not restricted | 2017 | 13.7 | 2 | Ate | 1200 mg Q3W | anti-PD-L1 | RECIST v1.1 | 49 |
|  |  |  |  |  |  |  |  | Chemo | - |  |  | 24 |
| Scott Gettinger (2016) ^49^ | NCT01454102  (Checkmate012) | I | NSCLC | IIIB/ IV | NA | 14.3 | 1 | Nivo | 3 mg/kg Q2W | anti-PD-1 | RECIST v1.1 | 52 |
| - ^50^ | NCT01450761 | III | SCLC | Extensive-Stage | 2012-2014 | 10.5 | 2 | Ipi + Chemo | 10 mg/kg Q3W | anti-CTLA-4 | mWHO | 566 |
|  |  |  |  |  |  | 10.2 |  | Chemo | - |  |  | 566 |
| - ^51^ | NCT01903993  (POPLAR) | II | NSCLC | IIIB/ IV or recurrent | 2013-2014 | - | 2 | Ate | 1200 mg Q3W | anti-PD-L1 | RECIST v1.1 | 144 |
|  |  |  |  |  |  |  |  | Chemo | - |  |  | 143 |
| Jonathan W. Goldman (2019) ^52^ | NCT02309177 | I | NSCLC | IIIB/ IV | 2015-2018 | 35.9 | 2 | Nivo (Concurrent) | 5mg/kg Q3W | anti-PD-1 | RECIST v1.1 | 22 |
|  |  |  |  |  |  | 30.7 |  | Nivo (Delayed) |  |  |  | 10 |
| Lyudmila Bazhenova (2019) ^53^ | NCT02785952  (Lung-MAP Sub-Study S1400I) | III | SCLC | IV | 2015-2018 | 17.4 | 2 | Nivo + Ipi | Nivo: 3 mg/kg Q2W Ipi: 1 mg/kg Q6W | Nivo: anti-PD-1 Ipi: anti-CTLA-4 | RECIST v1.1 | 125 |
|  |  |  |  |  |  |  |  | Nivo | 3 mg/kg Q2W | anti-PD-1 |  | 127 |
| Byoung C. Cho (2019) ^54^ | NCT03179436  (MK-1308-001) | I | SCLC | IIIB/ IV | NA | 11.0 | 1 | Mk-1308 + Pem | Mk-1308: 75 mg Q6W  Pem: 200 mg Q3W | anti-PD-1 | RECIST v1.1 | 40 |
| Luis Paz-Ares (2018) ^55^ | NCT02775435 | III | SCLC | IV | 2016-2017 | 7.8 | 2 | Pem + Chemo | 200 mg Q3W | anti-PD-1 | RECIST v1.1 | 278 |
|  |  |  |  |  |  |  |  | Chemo | - |  |  | 281 |
| Yusuke Okuma (2018) ^56^ | UMIN000020855/  UMIN000021734 | II | NSCLC | Advanced | 2016-2017 | NA | 1 | Nivo | 3 mg/kg Q2W | anti-PD-1 | RECIST v1.1 | 33 |
| Jong S. Lee (2018) ^57^ | NCT02175017 | II | NSCLC | IIIB/ IV or recurrent | 2014 | NA | 1 | Nivo | 3 mg/kg Q2W | anti-PD-1 | RECIST v1.1 | 100 |
| Stephen V. Liu (2018) ^58^ | NCT01633970 | Ib | NSCLC | IIIB/ IV or recurrent | NA | NA | 3 | Ate + Chemo1 | 1200 mg Q3W or 800 mg Q2W | anti-PD-L1 | RECIST v1.1 | 25 |
|  |  |  |  |  |  |  |  | Ate + Chemo2 |  |  |  | 25 |
|  |  |  |  |  |  |  |  | Ate + Chemo3 |  |  |  | 26 |
| Mark Socinski (2019) ^59^ | NCT02848651 | II | NSCLC | IIIB-IVB | NA | NA | 1 | Ate | 1200 mg Q3W or 800 mg Q2W | anti-PD-L1 | RECIST v1.1 | 152 |
| Toyoaki Hida (2017) ^60^ | JapicCTI-132072 | II | NSCLC | IIIB/ IV or recurrent | NA | NA | 1 | Nivo | 3 mg/kg Q2W | anti-PD-1 | RECIST v1.1 | 35 |
| Lecia Sequist (2016) ^61^ | NCT01375842 | I | SCLC | Extensive-Stage | NA | NA | 1 | Ate | 15 mg/kg or 1200 mg Q3W | anti-PD-1 | RECIST v1.1 | 17 |
| Narek Shaverdian (2017) ^62^ | NCT01295827  (KEYNOTE-001) | I | NSCLC | Locally Advanced or Metastatic | 2012-2014 | 32.5 | 4 | Pem + RT | 2 mg/kg or 10 mg/kg Q3W or 10 mg/kg Q2W | anti-PD-1 | irRC | 42 |
|  |  |  |  |  |  |  |  | Pem |  |  |  | 55 |
|  |  |  |  |  |  |  |  | Pem + Extracranial RT |  |  |  | 38 |
|  |  |  |  |  |  |  |  | Pem |  |  |  | 59 |
| Thomas J. Lynch (2012) ^63^ | NCT00527735  (CA184-041) | II | NSCLC | IIIB/IV | 2008-2009 | NA | 3 | Ipi + Concurrent Chemo | 10 mg/kg Q3W | anti-CTLA-4 | irRC | 70 |
|  |  |  |  |  |  |  |  | Ipi + Phased Chemo |  |  |  | 68 |
|  |  |  |  |  |  |  |  | Chemo |  |  |  | 66 |
|  |  |  |  |  |  |  |  | Ipi + Concurrent Chemo | 10 mg/kg Q3W | anti-CTLA-4 | mWHO | 70 |
|  |  |  |  |  |  |  |  | Ipi + Phased Chemo |  |  |  | 68 |
|  |  |  |  |  |  |  |  | Chemo |  |  |  | 66 |
| Martin Reck (2013) ^64^ | NCT00527737  (CA184-042) | II | SCLC | Extensive-Stage | 2008-2009 | NA | 3 | Ipi + Concurrent Chemo | 10 mg/kg Q3W | anti-CTLA-4 | irRC | 43 |
|  |  |  |  |  |  |  |  | Ipi + Phased Chemo |  |  |  | 42 |
|  |  |  |  |  |  |  |  | Chemo |  |  |  | 45 |
|  |  |  |  |  |  |  |  | Ipi + Concurrent Chemo | 10 mg/kg Q3W | anti-CTLA-4 | mWHO | 43 |
|  |  |  |  |  |  |  |  | Ipi + Phased Chemo |  |  |  | 42 |
|  |  |  |  |  |  |  |  | Chemo |  |  |  | 45 |
| Edurne Arriola (2016) ^65^ | NCT01331525 | II | SCLC | Extensive-Stage | 2011-2014 | NA | 1 | Ipi + Chemo | 10 mg/kg Q3W | anti-CTLA-4 | irRC | 53 |
| Shirish M. Gadgeel (2017) ^66^ | NCT02359019 | II | SCLC | Extensive-Stage | NA | NA | 1 | Pem | 200 mg Q3W | anti-PD-1 | irRC | 45 |
| M.D. Mattes (2019) ^67^ | - | II | NSCLC | Not restricted | NA | NA | 1 | ICI + RT | - |  | irRECIST | 34 |
| Anne C. Chiang (2020) ^68^ | NCT01375842 | I | SCLC | Relapsed/ Refractory | NA | NA | 1 | Ate | 15 mg/kg or 1200 mg Q3W | anti-PD-L1 | irRC | 17 |
|  |  |  |  |  |  |  |  |  |  |  | RECIST v1.1 | 17 |

NSCLC, non-small-cell lung cancer; SCLC, small-cell lung cancer; Chemo, chemotherapy; Ipi, ipilimumab, Nivo, nivolumab; Dur, durvalumab; Ate, atezolizumab; Tre, tremelimumab; Ave, avelumab; ICI, immune checkpoint inhibitors; RT, radiotherapy; QW, every week; Q2W, every 2 weeks; Q3W, every 3 weeks; Q4W, every 4 weeks; Q6W, every 6 weeks; NA, not available.
